# SLPI in Prostate Cancer

**DOI:** 10.3390/cancers18030487

**Published:** 2026-02-01

**Authors:** Dario Rosini, Irene Cosi, Pierpaolo De Iaco, Arcangelo Sebastianelli, Gioia Di Stefano, Sergio Serni, Gabriella Nesi, Rosario Notaro, Maria De Angioletti

**Affiliations:** 1 Laboratory of Cancer Genetics, Core Research Laboratory, Istituto per lo Studio, la Prevenzione e la Rete Oncologica (ISPRO), 50139 Florence, Italy; dario.rosini@hotmail.it (D.R.); irene.cosi90@gmail.com (I.C.); p.deiaco@student.unisi.it (P.D.I.); 2 Doctorate School GENETICS, ONCOLOGY and CLINICAL MEDICINE (GenOMeC), University of Siena, 53100 Siena, Italy; 3 Department of Experimental and Clinical Medicine, University of Florence, 50139 Florence, Italy; 4 Unit of Urological Minimally Invasive, Robotic Surgery and Kidney Transplantation, Careggi Hospital, University of Florence, 50139 Florence, Italy; 5 Histopathology and Molecular Diagnostic, Careggi University Hospital, 50139 Florence, Italygabriella.nesi@unifi.it (G.N.); 6 Department of Surgery and Translational Medicine, University of Florence, 50139 Florence, Italy; 7 Istituto di Chimica dei Composti Organometallici (ICCOM)—National Council of Research, Sesto Fiorentino, 50019 Florence, Italy

**Keywords:** androgen, androgen receptor, biomarker, ETS transcription factors, ETV1, ETV4, transgenic mouse model, prostate cancer, secretory leukocyte protease inhibitor, SLPI

## Abstract

Secretory Leukocyte Protease Inhibitor (SLPI) is a conserved serine protease inhibitor expressed on mucosal surfaces, which has multiple functions including anti-protease, anti-microbial and anti-inflammatory properties. SLPI plays critical roles in tissue homeostasis and pathology. Through its anti-protease ability, SLPI safeguards tissues from excessive damage caused by proteolytic enzymes released during inflammation and contributes to extracellular matrix remodeling, thereby influencing the cellular and tumor microenvironment. Furthermore, SLPI expression is implicated in shaping the immune landscape that facilitates tumor progression, and in driving epithelial–mesenchymal transition (EMT). Consequently, it is not surprising that SLPI plays a complex and context-dependent role across various malignancies. It is overexpressed in most cancers such as colorectal, gastric, pancreatic, and breast carcinomas, and this overexpression often correlates with a more advanced and aggressive disease. Conversely, its levels are reduced in head and neck squamous cell carcinoma and hepatocellular carcinoma, where elevated expression may be associated with a more favorable prognosis. This diverse behavior underscores that SLPI function in cancer is tissue-specific and dependent on the functional or pathological state. In prostate cancer, SLPI expression exhibits a bimodal behavior: levels are reduced in the early stages of the disease compared to normal tissues but become significantly upregulated in more advanced and aggressive stages of disease, with significantly higher levels observed in patients with castration-resistant prostate cancer. Elevated SLPI levels in prostate cancer correlate with a reduced prostate-specific antigen (PSA) progression-free survival. In this review, we outline the current evidence regarding the multifaceted functions of SLPI and its expanding role in cancer, focusing primarily on the recently described molecular mechanisms and clinical significance of SLPI in prostate carcinoma.

## Simple Summary

SLPI is a protein that usually acts as a protective shield for our body’s internal surfaces. Its main jobs are to prevent tissue damage, fight germs, and control inflammation. However, in the context of cancer, SLPI acts like a double-edged sword. While it normally keeps us healthy, many cancers—including lung and breast cancer—hijack this protein to grow and spread more easily. In these cases, high levels of SLPI often signal a more aggressive disease. Interestingly, the opposite happens in some cases, like liver cancer, where more SLPI can be a positive sign. Prostate cancer shows a unique pattern: SLPI protein levels are low in the early stages but rise sharply as the cancer becomes advanced and resistant to treatments. By studying these shifts, scientists can better understand how a tumor behaves, helping doctors predict the disease’s path and develop more effective, personalized treatments for patients.

## 1. Introduction

Prostate cancer is the most prevalent malignancy among men worldwide and ranks as one of the leading causes of cancer-related mortality in the male population [1,2]. It represents a global health concern because its prevalence continues to rise, largely driven by increasing life expectancy and population growth [3].

The risk of developing prostate cancer increases markedly with age, and over 85% of newly diagnosed patients are older than 60 [2,4]. Furthermore, prostate cancer is a highly heritable disease, as substantiated by twin studies [5]. Consequently, other established risk factors include a family history of cancer [6], ethnicity [7,8], and specific germline mutations, particularly in DNA repair genes (such as *BRCA1* and *BRCA2*) [9,10]. In addition, genome-wide association studies (GWAS) have identified over 450 single-nucleotide polymorphisms (SNPs) associated with prostate cancer risk [11,12,13,14]. While these SNPs effectively inform genetic risk prediction [8,15], the functional link between most of these variants and prostate tumorigenesis remains to be elucidated. Moreover, lifestyle and environmental factors such as smoking, obesity, diet, and low physical activity have been implicated, but conclusive evidence establishing a causal link is often lacking [16].

Prostate cancer is a highly heterogeneous disease, ranging from indolent, slow-growing tumors to aggressive, metastatic disease. In developed countries, the majority of cases are detected at an early localized stage, and are typically managed with active surveillance or curative approaches such as radical prostatectomy and radiotherapy [17,18]. However, a significant clinical challenge arises when the disease recurs or progresses. Treatment of advanced prostate cancer, including local relapse or systemic spread, relies primarily on androgen deprivation therapy, often combined with chemotherapy (e.g., taxanes) or novel agents targeting the androgen receptor (AR) pathway [10]. Despite initial treatment response, many patients develop castration-resistant prostate cancer (CRPC), the most aggressive and lethal form of the disease [10,19]. CRPC remains largely incurable, necessitating the continued search for novel therapeutic approaches, including immunotherapy and cancer vaccines [20].

Comprehensive genomic studies have identified numerous somatic genetic alterations that underlie prostate cancer heterogeneity [10] and inform the development of targeted therapeutic strategies [20]. Key alterations in prostate cancer include chromosomal translocations, most notably the fusion of *TMPRSS2* gene with members of the ETS transcription factor family (e.g., *ERG*, *ETV1*, *ETV4*), which occurs in more than 50% of cases [21,22,23,24]. These chromosomal translocations juxtapose the promoter of *TMPRSS2*, or another gene highly expressed in the prostate, to the coding region of an ETS gene, resulting in aberrant expression of the corresponding ETS transcription factor in prostate tissue [21,25]. Additional genetic alterations encompass mutations of *SPOP*, amplification of the *MYC* oncogene, and deletion and/or mutation of tumor suppressor genes like *PTEN* and *TP53* [10]. In advanced disease, mutations in DNA-repair genes, including *BRCA1* and *BRCA2*, are frequent, as are amplifications and activating mutation of the *AR* gene itself [26,27].

Beyond cell-intrinsic genomic changes, the tumor microenvironment (TME) is now recognized as a critical determinant of tumor initiation, progression, metastasis, and therapeutic response [28]. This paradigm also applies to prostate cancer [29,30]. The TME is a complex ecosystem in which the dynamic balance between secreted proteases and their inhibitors is essential for regulating extracellular matrix remodeling, modulating growth factor activation, and influencing cell signaling [31,32,33,34,35]. Secretory Leukocyte Protease Inhibitor (SLPI) is a well-known secreted protease inhibitor involved in regulating local inflammatory responses and tissue remodeling [36,37]. However, its precise role in prostate cancer progression and therapeutic resistance remains to be fully elucidated.

## 2. SLPI Structure, Function and Regulation

### 2.1. Structure

The secretory leukocyte protease inhibitor (SLPI) is one of the members of the Whey Acidic Protein (WAP) family, a small group of secreted proteins with a wide range of activities, notably the ability to inhibit proteases. WAPs are characterized by a conserved amino-acid domain, which contains eight cysteine residues forming four intramolecular disulfide bonds, named WAP domain (PFAM ID: PF00095) [38,39]. Investigation into SLPI dates back to 1965, when an acid-stable antiprotease activity was first identified in human seminal plasma [40] and later in various other tissues [41,42]. While this activity was initially attributed to different proteins, known by various tissue-specific names (e.g., antileukoproteinase, antileukoprotease, human seminal inhibitor), subsequent protein sequencing and gene cloning studies established that these were identical, representing a single protein encoded by a single gene [43,44,45,46].

**Figure 1:**
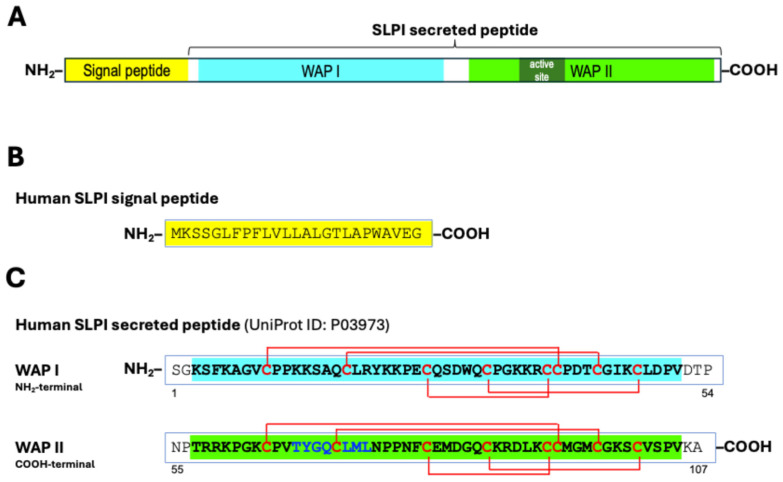
The structure of the human SLPI protein. (**A**) Structure of the native SLPI protein. The cartoon shows the signal peptide (yellow), the N-terminal WAP-I domain (cyan), and the C-terminal (green) WAP-II domains. (**B**) The amino acid sequence of the human SLPI signal peptide. (**C**) The amino acid sequence of the human secreted SLPI peptide. The N-terminal WAP-I domain is highlighted in cyan (above), and the C-terminal WAP-II domain is highlighted in green. The eight cysteine residues in each WAP domain are in red bold. The intramolecular disulfide bonds are indicated by red lines. The amino acid residues 67–74, which constitute the protease inhibitor functional site of SLPI, are highlighted in blue bold.

The human *SLPI* gene includes 4 exons and 3 introns spanning 2.6 kb mapped to chromosome 20q13.12 [44] within a rapidly evolving gene region on chromosome 20q13, where most of *WAP* genes are tightly clustered (*WAP*/*WFDC* locus), suggesting that they originate through the duplication of a common ancestor [47,48]. The human *SLPI* gene encodes a 132 amino-acid polypeptide chain (Figure 1A) composed of a signal peptide and of a secreted peptide (Figure 1B,C) [43,44]. The secreted peptide is an 11.7 kDa cysteine-rich, non-glycosylated protein composed of 107 amino acids, arranged in a single polypeptide chain that contains two homologous WAP domains (UniProt ID: P03973) (Figure 1C) [43,44]. The exon organization mirrors its modular structure with each functional domain encoded by a separate exon [44]. The *SLPI* gene is evolutionarily conserved, with human and murine *SLPI* exhibiting an overall 68% homology at the nucleotide level and 60% at the amino acid level, specifically showing 80% identity in the signal peptide and 52% identity in the secreted protein, where all 16 cysteine residues are preserved [49].

The anti-protease activity of WAPs is exerted, as in Kunitz and Kazal-type inhibitors, by a non-covalent binding to the catalytic cleft of the protease to block substrate binding [50,51]. The 3D structure of SLPI protein shows two well-separated domains arranged in a boomerang-like shape [52]. In addition, mutational and structural studies clearly demonstrated that SLPI protease inhibitor function resides mainly in the WAP II (C-terminal) domain [52,53,54,55], specifically, between residues 67–74 (Figure 1C) [54,56]. The biological function of the SLPI N-terminal WAP I domain is less clear; however, it is thought to be responsible for SLPI antimicrobial function, although the intact molecule exhibits the full antimicrobial function [57,58,59].

### 2.2. SLPI Expression and Physiological Function

The *SLPI* gene is expressed across a wide variety of human cell types [60,61,62,63]. Under physiological conditions, SLPI mRNA and protein are primarily expressed by epithelial cells lining mucosae and skin, fulfilling its roles in local defense and homeostasis. Specifically, SLPI is highly expressed at body barriers, including the nasal, bronchial, and alveolar epithelia, the gastrointestinal and genitourinary mucosae, and the ductal cells of the salivary glands [64,65,66]. SLPI is also expressed by epidermal keratinocytes and by acinar and secretory epithelial cells of organs such as the cervix, seminal vesicles, and prostate [62,67]. Consequently, SLPI is primarily a secreted product found in body fluids at mucosal surfaces, where its concentration is significantly higher than in plasma [68]. However, SLPI is also constitutively or transiently expressed in blood cells (monocytes, macrophages, granulocytes, dendritic cells, and platelets) [69,70,71,72], and in brain cells (neurons and astrocytes) [73].

The primary and most extensively characterized physiological role of SLPI is the reversible inhibition of Neutrophil Elastase (NE) [42]. In the upper respiratory tract secretions, SLPI accounts for up to 97% of the NE inhibitory capacity [74], thereby protecting tissue proteins and the extracellular matrix from degradation by NE released during inflammatory processes and ensuring proper resolution of inflammation [42,74]. SLPI also inhibits other serine proteases, such as chymotrypsin, cathepsin G, and trypsin, though with lower affinity compared to NE [36,43,56,75,76].

Beyond its direct enzymatic control, SLPI exhibits crucial secondary, non-protease inhibitory mechanisms that operate at both the extracellular and intracellular levels [77,78,79]. It is also notable that secreted SLPI is able to enter cells, localizing to the cytoplasm and nucleus, where it may exert most of its intracellular functions [80,81]. SLPI suppresses the expression of matrix metalloproteinases (MMPs), such as MMP1 and MMP9, in monocytes [80]. Intracellularly, SLPI is a critical modulator of inflammation by regulating several signaling pathways—including NF-kB, ERK, and transforming growth factor-beta (TGF-β)—and suppressing the synthesis of pro-inflammatory mediators like tumor necrosis factor-alpha (TNFα), IL8 and nitric oxide by various immune cells like monocytes, dendritic cells, and macrophages [49,80,82,83,84]. SLPI immunomodulatory effects also extend to adaptive immunity, suppressing CD4 T cells via monocytes and dendritic cells [71,85], and inhibiting immunoglobulin class switching in activated B cells by targeting NF-kB in tonsillar epithelial cells [86]. Furthermore, SLPI plays a role in the proliferation and differentiation of human myeloid cells [81], and it plays a role in wound healing in mice [87,88], and possibly also in humans [89,90,91].

Furthermore, SLPI exhibits significant antimicrobial activity against bacteria and fungi [57,92,93,94,95]. It is also implicated in the reduction in HIV-1 transmission via mucosal secretions [66,96,97,98].

## 3. SLPI in Cancer

The ability of SLPI to inhibit extracellular matrix proteolysis and to dampen inflammation, two central processes in cancer development and progression, suggests a role as a tumor suppressor. Consistently, SLPI is underexpressed in a minority of cancers, including head and neck squamous cell carcinoma, oral squamous cell carcinoma, hepatocellular carcinoma, and nasopharyngeal carcinoma [99,100,101,102]. However, paradoxically SLPI is overexpressed in the majority of solid tumors—including pancreatic, papillary thyroid, endometrial, ovarian, non-small lung carcinoma, colorectal, and gastric cancer—where its high expression often correlates with poor prognosis and advanced disease (reviewed in [37,103,104]). Recently, SLPI overexpression has also been observed in hematological malignancies, such as acute myeloid leukemia (AML), where it is overexpressed in leukemic myelocytes [105] and elevated levels are present in the bone marrow plasma of AML patients [106].

Thus, SLPI exerts multiple, highly complex, and tissue-specific roles via diverse/multiple mechanisms [37]. The contribution of SLPI to tumorigenesis extends beyond its classical anti-protease activity, involving non-proteolytic mechanisms that reshape the tumor microenvironment (TME). SLPI promotes cell invasion and migration through the induction of matrix metalloproteinases (specifically MMP2 and MMP9) [83,107,108]. Moreover, SLPI drives angiogenesis and vascular mimicry—key processes for nutrient supply and tumor growth—by modulating the expression of factors such as vascular endothelial growth factor (VEGF) [109,110]. Furthermore, SLPI facilitates the epithelial–mesenchymal transition (EMT), resulting in the loss of epithelial markers and the acquisition of a migratory and invasive mesenchymal phenotype [111]. In lung adenocarcinoma specifically, a cancer cell sub-population expressing SLPI drives invasion via EMT and cooperates with specific tumor-associated macrophages (TAM) to generate an immunosuppressive niche at tumor leading edges [112].

Beyond cancer cells, SLPI-expressing macrophages have been identified in several cancers co-localizing with a sub-population of cancer-associated fibroblasts (CAFs) at the border between tumors and healthy tissue, potentially preventing immune infiltration and resulting in poor prognosis [113]. Similarly, human mesenchymal stem cells from AML patients secrete SLPI that modulates the gene expression profile of hematopoietic stem cells—upregulating cell cycle markers and downregulating tumor suppressors—suggesting that the AML niche leads to altered SLPI secretion, which in turn plays a role in leukemic progression [106].

At the intracellular level, SLPI regulates gene expression by translocating to the nucleus and interacting with NF-κB [78,114] and the Akt/PI3K pathway [101,115,116].

The ability of SLPI to promote cancer cell growth and survival has been reported for several cancers, including colorectal, endometrial, gastric, ovarian, pancreatic, and papillary thyroid cancers [101,117,118,119,120,121]. SLPI overexpression drives cell growth and survival by protecting the epithelial growth factor progranulin from degradation in ovarian cancer cells [122,123]. In addition, SLPI overexpression has been shown to protect against apoptosis in several solid tumors—including colorectal, endometrial, gastric, and pancreatic cancer [117,118,119,120,124]—as well as leukemias [106].

Conversely, in a subset of cancers, SLPI induces apoptosis and acts as a tumor suppressor. It is intriguing that these pro-apoptotic functions have been documented mostly in cellular models of cancers where SLPI is underexpressed, such as head and neck squamous cell carcinoma, oral squamous cell carcinoma [125,126], and hepatocellular carcinoma [127]. This complexity is further mirrored in oral squamous cell carcinoma, where conflicting reports describe SLPI as anti-tumorigenic because it is underexpressed and interferes with tumor invasion and metastasis [100], but also as essential for invasion via MMP interaction in a myoma in vitro model of invasion [128,129]. This highlights the critical influence of the experimental model and microenvironmental context on SLPI function.

These observations confirm the pleotropic nature of SLPI, which acts as a “double-edged sword” dependent not only on tissue specificity but also on tumor stage. This intriguing dichotomy is particularly evident in breast and colorectal cancers. The role of SLPI in breast cancer is ambivalent, as either decreased or increased expression levels have been correlated with either cancer progression or regression [130,131,132,133,134]. Specifically, high SLPI levels drive metastasis and poor survival in triple-negative breast cancer [108,135,136], whereas recent findings suggest that in non-metastatic basal subtypes, SLPI acts as a favorable prognostic marker [136]. This supports the hypothesis that SLPI may exert protective, anti-invasive effects in early-stage tumors while fueling progression in metastatic contexts.

A similar dichotomy is observed in colorectal cancer. High SLPI expression in liver metastases correlates with worse outcomes [137]. However, in Stage III patients with microsatellite stable tumors, high SLPI expression is associated with reduced disease recurrence and a better response to adjuvant chemotherapy [138]. This suggests that SLPI influence shifts from beneficial (predicting chemo-response/inhibiting recurrence) in localized disease to detrimental (promoting colonization) once distant metastases are established [138].

The expression pattern of SLPI is even more peculiar in prostate cancer, where it exhibits a unique biphasic pattern characterized by reduced levels in early-stage disease [139] and increased levels during progression, such as in metastatic CRPC patients [140,141].

## 4. SLPI in Prostate and Prostate Cancer

### 4.1. Physiological Distribution and Role of SLPI in Male Genitourinary System

Under physiological conditions, SLPI is expressed by secretory epithelial cells throughout the male reproductive tract and secreted into the seminal fluid. Immunostaining has detected high levels of SLPI protein within the seminal vesicles [142], with comparatively lower levels localized to the epithelial cells of the prostate, epididymis, and the apical germinal epithelium of the testis [62]. Although the precise physiological role of SLPI in the male genitourinary tract remains to be fully elucidated, its high levels in seminal plasma, approximately 20 mg/L [142], underscores its critical function in protecting both glandular tissue and sperm cells from proteolytic damage, inflammation, and infections. Specifically, it has been hypothesized that SLPI safeguards sperm cells against the degradative action of leukocyte proteases, while notably it exerts no inhibitory control over the prostate-specific antigen (PSA), the most abundant protease found in seminal fluid [62].

### 4.2. SLPI Expression in Prostate Cancer

Early transcriptomic investigations utilizing whole-genome cDNA microarrays identified *SLPI* as one of 32 genes that consistently exhibit a distinct expression gradient across genitourinary tissues: levels are highest in the seminal vesicles, lower in the normal prostate, and lowest in prostate cancer [139,143]. This expression gradient was further confirmed at the protein level by immunohistochemistry on tissue microarrays, which also revealed both cytoplasmic and nuclear localization of the SLPI protein within the cells of these tissues [139]. The disparity between the high SLPI levels in the seminal vesicles—a tissue with a remarkably low cancer incidence—and the low levels in the prostate suggested that SLPI may exert a protective effect against oncogenesis. Mechanistically, it has been hypothesized that the downregulation of SLPI in the prostate creates a microenvironment permissive to chronic inflammation, an established etiological factor in prostate carcinogenesis, which may contribute to the development of prostate cancer [139].

**Figure 2:**
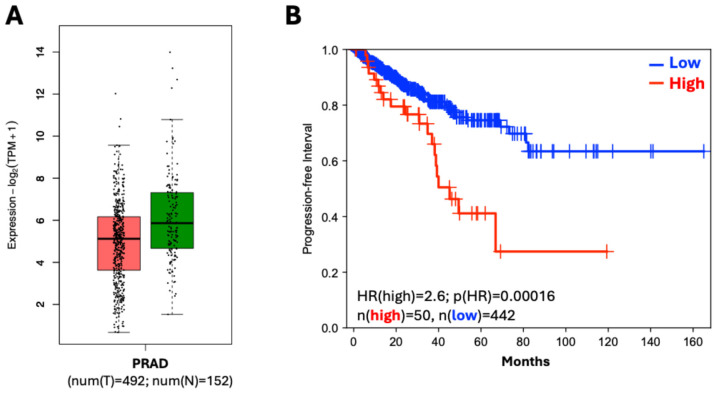
TCGA analysis. (**A**) Comparison of *SLPI* expression levels of tumor (T) versus normal tissues (N) in prostate. Data from The Cancer Genome Atlas (TCGA) Prostate Adenocarcinoma (PRAD) cohort and from the CGA TARGET GTEx dataset. Boxplot generated by http://gepia2.cancer-pku.cn/ (URL accessed on 10 December 2025). (**B**) Disease-Free Survival (DFS) of patients with high *SLPI* expression (≥90% percentile) versus patients with intermediate or low expression (<90% percentile). Kaplan-Meyer curve generated by http://gepia2.cancer-pku.cn/ (URL accessed on 10 December 2025).

These observations have been corroborated by high-throughput data. *SLPI* was identified as a differentially expressed gene in prostate cancer through RNA-seq analysis of transcriptomic data from The Cancer Genome Atlas (TCGA) Prostate Adenocarcinoma (PRAD) cohort [144,145,146,147]. Specifically, these bioinformatic studies found that *SLPI* mRNA levels were significantly lower in tumoral compared to normal prostate tissues (Figure 2A). These results were further validated using immunohistochemistry data from the Human Protein Atlas (HPA), which demonstrated that SLPI protein expression is markedly higher in normal prostate tissue than in malignant counterparts [144,145].

The biological relevance of SLPI downregulation is underscored by experimental evidence from transgenic murine models. Specifically, the prostate-specific expression of a truncated human *ETV4* transgene leads to the development of murine prostatic intraepithelial neoplasia in approximately two-thirds of these ETV4 transgenic mice by 10–11 months of age [148]. In this model of early-stage disease, SLPI is significantly reduced at both the mRNA and protein levels, mirroring the patterns observed in human patients and suggesting a potential regulatory link between ETS transcription factors and SLPI downregulation [111].

Consistent with these observations, several prostate cancer cell lines—including DU145, VCaP, LNCaP, C4-2, 22Rv1, and LAPC4—show minimal SLPI expression at both the mRNA and protein levels [140]. In addition, SLPI levels in these lines, as well as in the androgen-independent PC3 line, are significantly lower than those found in the immortalized normal prostate epithelial cell line, RWPE [111]. However, a marked phenotypic shift occurs in a subset of castration-resistant (CRPC) models; in fact, SLPI expression is markedly elevated in the androgen-dependent LNCaP prostate cell line derivative, such as the bone-metastatic, androgen-independent C4-2B line [140], as well as in the enzalutamide-resistant AILNCaP14 and AILNCaP15 lines [141]. The overexpression of SLPI in these CRPC cellular models suggests a functional shift during disease progression, challenging the initial characterization of SLPI as a tumor suppressor. The phenotypic shift in cellular models is corroborated by transcriptomic analyses of public microarray data sets [149,150], which reveal significant *SLPI* overexpression in metastatic lesions compared to primary tumors [140]. These findings are supported by Miyazaki et al., who reported that 44.8% of 154 radical prostatectomy specimens were SLPI-positive, with positivity being an independent risk for a significantly shorter post-surgery PSA progression-free survival [141]. In contrast, some analyses of the TCGA-PRAD cohort suggested that elevated *SLPI* correlates with improved Overall Survival (OS); however, these conclusions are likely a statistical artifact due to the extreme paucity of mortality events (only 10 deaths) and a short follow-up period, compared with the long natural history of prostate cancer [151]. Indeed, when evaluating Disease-Free Survival (DFS)—a more reliable endpoint for this cohort—patients with high *SLPI* expression exhibited significantly shorter survival than those with intermediate or low expression (Figure 2B), in direct concordance with the findings of Miyazaki et al. [141].

Importantly, the expression patterns observed in prostate tissue are mirrored by the circulating SLPI concentration in the serum. In a cohort of 107 patients with prostate cancer and 20 healthy controls, serum SLPI concentrations were slightly lower in patients with localized prostate cancer (median 47.18 ng/mL) than in healthy individuals (55.77 ng/mL) [140]. While levels in metastatic hormone-naïve patients remained comparable to those of normal controls, concentrations were significantly elevated in advanced disease—specifically in patients with metastatic castration-resistant prostate cancer [140]. A similar trend for serum SLPI was observed in a separate cohort of 69 patients encompassing benign prostatic hyperplasia and various stages of prostate cancer; however, statistical significance was not reached, likely due to the limited sample size [141].

Taken together, these data delineate a complex scenario in which—nearly uniquely among malignancies—prostate cancer exhibits a distinct biphasic pattern of SLPI expression. This trajectory is characterized by reduced levels during the early stages of the disease [139], followed by increased levels during the progression to advanced disease [140,141]. Such a peculiar expression profile suggests that SLPI plays a multifaceted role in prostate cancer pathogenesis, potentially shifting from a tumor-suppressive to an oncogenic function. In this context, it is noteworthy that the *SLPI* gene is located at 20q13.12 within the HPC20 locus, a genomic region long associated with prostate cancer susceptibility [152,153,154].

### 4.3. SLPI as an Oncogenic Driver in Prostate Cancer

The overexpression of SLPI in advanced prostate cancer indicates that it acts more as an oncogene than as a tumor suppressor. This oncogenic role is corroborated by several silencing and overexpression studies in cellular model of prostate cancer. *SLPI* silencing in the androgen-independent C4-2B cells reduces cell growth, anchorage-independent colony formation, as well as cell invasion. Interestingly, while SLPI overexpression in the parental androgen-dependent LNCaP cells does not significantly increase proliferation in vitro, it markedly enhances in vivo tumor growth, leading to larger tumors when subcutaneously injected into castrated immunodeficient NOD/SCID mice [140]. Mechanistically, transcriptomic analysis of C4-2B cells reveals that *SLPI* silencing activates p53-dependent apoptotic pathways while simultaneously downregulating multiple anti-apoptotic mediators. Functional assays further demonstrate that C4-2B cells exhibit reduced sensitivity to TNFα-mediated apoptosis compared to their parental androgen-dependent LNCaP cells. This resistant phenotype is directly linked to SLPI levels: overexpressing SLPI in LNCaP cells confers resistance to TNFα, whereas *SLPI* silencing in C4-2B cells restores sensitivity to TNFα [140]. By inhibiting TNFα-mediated apoptosis, the primary driver of prostate regression following castration [155,156], SLPI enables cancer cells to persist under the selective pressure of androgen deprivation. This adaptive mechanism directly contributes to the development of therapy resistance and disease progression.

Furthermore, evidence from LNCaP cells indicates that SLPI physically interacts with the epithelial growth factor progranulin (PGRN), shielding it from elastase-mediated degradation. This protection of a factor involved in cell survival and proliferation, combined with the anti-apoptotic effects of SLPI, likely contributes to the enhanced growth observed in advanced disease [140].

Beyond cancer cell lines, silencing and overexpression experiments in the immortalized normal prostate line, RWPE, confirm that SLPI impairs apoptosis while promoting migration and invasion. Specifically, in this model, SLPI upregulates MMP expression and promotes the E-cadherin/N-cadherin switch, thereby inducing EMT and the acquisition of a migratory mesenchymal phenotype [111]. Additionally, SLPI has been shown to drive vascular mimicry in prostate cells, a process that further facilitates tumor invasion and metabolic adaptation [110].

Finally, recent studies have identified an expanded role for SLPI that extends beyond its direct effects on cancer cells to encompass the modulation of the tumor immune microenvironment. Bioinformatic analyses of public transcriptomic datasets have correlated SLPI expression with increased infiltration of gamma-delta T cells [145], resting dendritic cells, CD8 T cells, and resting regulatory T cells, as well as reduction in M2-polarized macrophages [147]. However, none of these correlations have been directly validated in either in vitro or in vivo functional models.

**Figure 3:**
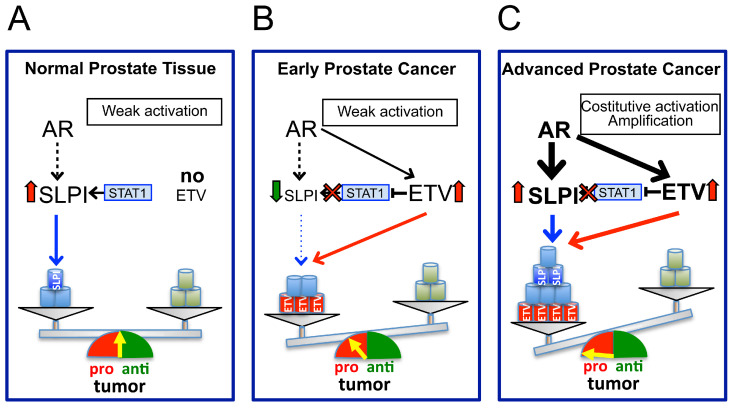
Regulation of SLPI levels through the interplay between the ETS transcription factors and the Androgen/Androgen Receptor axis and its effects on the neoplastic phenotype of prostate cancer. (**A**) Normal prostate. Under physiological conditions, ETS transcription factors are not expressed in healthy prostate tissue. Basal SLPI expression is maintained via positive regulation by the Androgen Receptor (AR) and STAT1. In this physiological context, basal levels of SLPI support homeostatic cellular functions, including regulated migration, epithelial–mesenchymal transition (EMT), and anti-apoptotic signaling. (**B**) Early-stage prostate cancer. The onset of the androgen-driven ETV4 or ETV1 expression promotes initial pro-tumoral effects; however, these ETS factors also trigger STAT1 downregulation, leading to reduced SLPI expression. This SLPI reduction dampens its contribution to migration, EMT, and apoptosis evasion abilities of prostate cells. Consequently, this mitigates the pro-tumorigenic effects of ETV4/ETV1 and, ultimately, contributes to the typically indolent clinical phenotype of early-stage prostate cancer. (**C**) Advanced prostate cancer. During progression, constitutive AR activation or gene amplification drives the upregulation of both ETV4/ETV1 and SLPI, because AR-mediated upregulation of SLPI overcomes the inhibitory effects of ETV4/ETV1 on SLPI expression. Thus, the resulting elevated SLPI levels act synergistically with ETS factors to bolster the malignant phenotype of the prostate cancer cells, fostering more aggressive cancer behavior. **ETV** refers to ETV4 or ETV1. The arrows connecting the proteins indicate positive regulation (activation), while the “x” symbols represent inhibition. **Line weight** represents the relative strength of the regulatory effect. **Font size** indicates the relative expression level of the indicated factors. **Large Vertical arrows** denote increases (upward) or decreases (downward) in expression. The yellow arrow serves as the hand on the dial scale, representing the dynamic balance between pro- and anti-tumor activities. Reproduced from Cosi et al. Biochim Biophys Acta Mol Basis Dis 2025, 1871, 167975 [111].

Focusing strictly on the functional experimental evidence, the concordant data from *SLPI* silencing and overexpression experiments across various models support the view that in the prostate, as in other malignancies, SLPI functions as an oncogene. However, this oncogenic role may appear paradoxical given its biphasic expression pattern, which at first glance suggests a functional shift from tumor-suppressive to tumor-promoting roles. Such a pattern resembles that of transforming growth factor-beta (TGF-β), which, in various tumors—including prostate cancer—acts as a tumor suppressor in early stages and as a driver of malignancy in advanced disease [157,158,159,160]. However, we have recently proposed that for SLPI this paradox is merely apparent and that, unlike TGF-β, SLPI does not undergo a functional “switch” [111]. Rather, the biological impact of SLPI is dictated by its absolute expression levels. In early-stage prostate cancer, SLPI is expressed below physiological levels. As demonstrated in *SLPI*-silenced cells, these sub-physiological levels impair critical neoplastic traits—including migratory capacity, invasive potential, and apoptosis evasion—thereby contributing to the characteristic indolent phenotype and slow progression of early disease. Conversely, when SLPI is upregulated—as in RWPE cells overexpressing SLPI and in patients with advanced prostate cancer—the levels of SLPI above the physiological baseline actively drive the malignant phenotype and accelerate disease progression. Consequently, the apparent transition from tumor suppression to promotion is a quantitative rather than a qualitative phenomenon: low levels hinder neoplastic transformation, while high levels fuel it (Figure 3) [111].

### 4.4. Regulation of SLPI in Prostate Cancer

The reduced levels of SLPI in prostate cancer were initially attributed to transcriptional silencing via promoter hypermethylation, a hallmark of tumor suppressor genes [139], because SLPI expression is increased in prostate cancer cells treated with the demethylating agent 5-aza-2′-deoxycytidine (decitabine) [161]. However, this response was observed only in the MDA-PCa-2b cell line and not in LNCaP, DU145, or PC3 cells, suggesting that hypermethylation-mediated *SLPI* silencing may be restricted to a specific molecular subset of prostate cancers.

Another proposed mechanism for SLPI downregulation involves the Androgen Receptor (AR) acting through an indirect microRNA-mediated pathway. Specifically, the AR, in coordination with other transcription factors, upregulates miR-525-5p, which subsequently inhibits SLPI expression [110]. However, this mechanism was demonstrated only in AR-negative PC3 cells following ectopic *AR* transfection, calling its physiological relevance in endogenous AR-positive systems into question. Conversely, evidence from castration-resistant models indicates that in C4-2B cells, SLPI is positively regulated by the AR in a ligand-independent manner [140,162].

Furthermore, the reduced SLPI levels observed in ETV4 transgenic mice, which develop premalignant prostate lesions, mirror the expression patterns found in early-stage human prostate cancer, suggesting that ETV4 functions as a negative regulator of SLPI [111]. This hypothesis is supported by in vitro studies: *ETV4* silencing in DU145 and PC3 cell lines, or its overexpression in normal RWPE cells, consistently shows that ETV4 downregulates SLPI. Consistent with these findings, similar silencing and overexpression experiments in LNCaP and RWPE cell lines demonstrate that ETV1—which, like ETV4, is a member of the PEA3 subfamily of ETS transcription factors—also mediates the downregulation of SLPI, mirroring the effects observed with ETV4. Together, these findings identify the ETS factors, ETV4 and ETV1, as drivers of SLPI downregulation via an indirect mechanism, likely mediated by the downregulation of STAT1 [111], a known positive regulator of SLPI [163].

We have proposed a mechanism to explain how this regulation results in a biphasic expression pattern [111], based on the fact that both ETS and SLPI expression are regulated by the androgen/AR axis [140]. During the early stages of prostate cancer, the androgen/AR axis drives the overexpression of ETS transcription factors, ETV4 and ETV1, which in turn downregulate SLPI, effectively overriding any AR-mediated SLPI upregulation. In contrast, during disease progression, the AR amplification or its constitutive activation—a molecular hallmark occurring in over 80% of patients with advanced prostate cancer [164], appears to override the SLPI downregulation mediated by ETS factors, leading to its subsequent elevation. The crosstalk between these regulatory pathways, which exert opposing influences on SLPI expression, accounts for the shift between low and elevated SLPI levels observed across the different stages of prostate cancer (Figure 3) [111]. However, this regulatory mechanism has an experimental basis only in prostate cancer cases characterized by the overexpression of ETS factors, such as ETV4 or ETV1. Consequently, further investigations are required to determine whether similar mechanisms exist in tumors driven by other common genetic alterations, such as the ERG fusion, or to identify the alternative pathways responsible for SLPI modulation in ETS-negative disease.

## 5. Perspectives

The biphasic expression of SLPI underscores its profound biological relevance, as both its reduced and elevated levels significantly influence the clinical and biological evolution of prostate cancer. Consequently, SLPI represents a significant herald of disease evolution; its distinct shift from early-stage downregulation to late-stage overexpression directly mirrors the transition from an indolent to an aggressive phenotype. This alignment with disease progression highlights the potential utility of SLPI as a prognostic biomarker. Specifically, monitoring serum SLPI concentrations could serve as a valuable adjunct to PSA testing, providing a more comprehensive biochemical profile of the transition toward androgen independence. Nevertheless, the precise relationship between SLPI levels, therapy response, and long-term clinical outcomes remains a subject of active investigation and necessitates validation in larger prospective cohorts [165]. Finally, the role of SLPI in prostate cancer exemplifies the intricate and essential interplay between genetic and microenvironmental factors. Understanding how these mutual interactions shape the evolutionary trajectory of the tumor is vital for refining our ability to predict, monitor, and ultimately intercept disease progression.

## Data Availability

No new data were created or analyzed in this study. Data sharing is not applicable to this article.

## Abbreviations

The following abbreviations are used in this manuscript:

AML	Acute Myeloid Leukemia
AR	Androgen Receptor
BRCA1	BRCA1 DNA Repair Associated
BRCA2	BRCA2 DNA Repair Associated
CAFs	Cancer-Associated Fibroblasts
CRPC	Castration Resistant Prostate Cancer
EMT	Epithelial-to-Mesenchymal Transition
ETS	E26 Transformation specific, Erythroblast Transformation Specific
ERG	ETS-related gene
ETV1	ETS Variant gene 1
ETV4	ETS Variant gene 4
GWAS	Genome-Wide Association Studies
HIV-1	Human Immunodeficiency Viruses 1
IL8	Interleukin 8
MMP	Metalloproteinase
MYC	MYC Proto-Oncogene, BHLH Transcription Factor
NE	Neutrophil Elastase
NF-kB	Nuclear Factor kappa-light-chain-enhancer of activated B cells
OSCC	Oral Squamous Cell Carcinoma
PSA	Prostate-Specific Antigen
PTEN	Phosphatase and Tensin Homolog
SLPI	Secretory Leukocyte Protease Inhibitor
SNPs	Nucleotide Polymorphisms
SPOP	Speckle Type BTB/POZ Protein
TAM	Tumor Associated Macrophages
TGF-beta	Transforming Growth Factor Beta
TME	Tumor Microenvironment
TMPRSS2	Transmembrane Protease, Serine 2
TNF-alpha	Tumor Necrosis Factor alpha
TP53	Tumor Protein P53
VEGF	Vascular Endothelial Growth Factor
WAP	Whey Acidic Protein
